# Using DNA Metabarcoding to Identify the Floral Composition of Honey: A New Tool for Investigating Honey Bee Foraging Preferences

**DOI:** 10.1371/journal.pone.0134735

**Published:** 2015-08-26

**Authors:** Jennifer Hawkins, Natasha de Vere, Adelaide Griffith, Col R. Ford, Joel Allainguillaume, Matthew J. Hegarty, Les Baillie, Beverley Adams-Groom

**Affiliations:** 1 National Botanic Garden of Wales, Llanarthne, Carmarthenshire, United Kingdom; 2 School of Pharmacy and Pharmaceutical Sciences, Cardiff University, Cardiff, United Kingdom; 3 Institute of Biological, Environmental and Rural Sciences, Aberystwyth University, Aberystwyth, United Kingdom; 4 Department of Biological, Biomedical and Analytical Sciences, University of the West of England, Bristol, United Kingdom; 5 National Pollen and Aerobiology Research Unit, University of Worcester, Worcester, United Kingdom; University of Milano Bicocca, ITALY

## Abstract

Identifying the floral composition of honey provides a method for investigating the plants that honey bees visit. We compared melissopalynology, where pollen grains retrieved from honey are identified morphologically, with a DNA metabarcoding approach using the *rbcL* DNA barcode marker and 454-pyrosequencing. We compared nine honeys supplied by beekeepers in the UK. DNA metabarcoding and melissopalynology were able to detect the most abundant floral components of honey. There was 92% correspondence for the plant taxa that had an abundance of over 20%. However, the level of similarity when all taxa were compared was lower, ranging from 22–45%, and there was little correspondence between the relative abundance of taxa found using the two techniques. DNA metabarcoding provided much greater repeatability, with a 64% taxa match compared to 28% with melissopalynology. DNA metabarcoding has the advantage over melissopalynology in that it does not require a high level of taxonomic expertise, a greater sample size can be screened and it provides greater resolution for some plant families. However, it does not provide a quantitative approach and pollen present in low levels are less likely to be detected. We investigated the plants that were frequently used by honey bees by examining the results obtained from both techniques. Plants with a broad taxonomic range were detected, covering 46 families and 25 orders, but a relatively small number of plants were consistently seen across multiple honey samples. Frequently found herbaceous species were *Rubus fruticosus*, *Filipendula ulmaria*, *Taraxacum officinale*, *Trifolium* spp., *Brassica* spp. and the non-native, invasive, *Impatiens glandulifera*. Tree pollen was frequently seen belonging to *Castanea sativa*, *Crataegus monogyna* and species of *Malus*, *Salix* and *Quercus*. We conclude that although honey bees are considered to be supergeneralists in their foraging choices, there are certain key species or plant groups that are particularly important in the honey bees environment. The reasons for this require further investigation in order to better understand honey bee nutritional requirements. DNA metabarcoding can be easily and widely used to investigate floral visitation in honey bees and can be adapted for use with other insects. It provides a starting point for investigating how we can better provide for the insects that we rely upon for pollination.

## Introduction

Insect pollination is a key regulating ecosystem service, vital to the functioning of terrestrial habitats. It is also crucial to crop production systems with 75% of crop species benefiting from insect pollinators [1]. The honey bee (*Apis mellifera*) is of major importance as a pollinator of both wild and crop plants [2]. It is also the provider of honey, a high-value nutritional product [2, 3]. Numbers of managed honey bee colonies have decreased substantially in Europe and North America, although this has been balanced by increases in countries such as China and Argentina [4]. Overall managed honey bee colonies have increased by around 45% between 1947 and 2005, but in the same time period the proportion of crops requiring insect pollination has increased threefold, meaning food production is more dependent on insect pollinators than ever before [4].

Although the number of managed honey bee colonies has increased, there has been considerable concern worldwide over increased rates of honey bee colony loss due to poor health, in particular due to the syndrome described as Colony Collapse Disorder (CCD) [5].

It is being increasingly recognised that there are a number of interacting drivers causing poor health and colony loss in honey bees. Pests and diseases, exposure to agrochemicals, apicultural mismanagement and lack of genetic diversity all play a part [2, 3]. Alongside this are reductions in habitat suitable for foraging. Honey bee nutritional needs are met by nectar, pollen and water [6]. Intensive farming practices lead to reductions in habitats with diverse floral resources and increases in mass-flowering crops that provide a single floral resource over a limited period of time [1, 2]. Pollen from different plant species varies greatly in its protein content along with the composition of lipids, carbohydrates, vitamins and minerals [7]. Nectar varies in the type and concentration of sugar and also contains a range of other trace elements [6]. Pollen diversity and quantity is known to affect disease tolerance and longevity in honey bees although further research is required in this area [8–12]. It is therefore a distinct possibility that nutritional stress due to lack of suitable foraging habitat may combine with other factors to cause ill health and colony loss in honey bees [1, 7, 13, 14].

Recommendations for improving honey bee health, as well as maintaining populations of wild pollinators, include reducing exposure to insecticides, preventing and limiting the spread of disease and providing a greater diversity of floral resources throughout the year [1]. In farmed landscapes greater floral resource can be achieved through maintaining areas of semi-natural habitat, sowing flower-rich field margins and creating and maintaining hedgerows [1].

Urban areas can potentially provide valuable foraging resources for honey bees and wild pollinators [15]. Planting appropriate plants in gardens and amenity spaces can provide a high diversity of floral resources and there are a number of schemes that provide lists of ‘pollinator friendly’ plants [16–19]. Whilst some of these lists are based on careful observation over many years, others are based on anecdotal information and generally lack a firm evidence base [20]. In order to optimise the provision of appropriate floral resources, a greater understanding is required of the foraging preferences of honey bees and wild pollinators and how this relates to their nutritional needs [1, 7, 21].

Foraging in honey bees has been investigated in a number of ways. The pioneering work of Karl von Frisch and his associates [22] led to the discovery that the ‘waggle dance’ of honey bees indicates the direction and distance to resources. A number of studies have decoded the honey bee waggle dance to look at distances travelled, but these studies have not tended to focus on the plants visited [23–27]. Similarly, honey bees can be fitted with RFID tags that track movement, but again floral visitation has not been monitored [28].

Methods used to assess floral visitation include identifying the pollen returned to the hive by honey bees, either directly by collecting pollen at the hive entrance using ‘pollen traps’ or through examining pollen found within honey [29, 30]. Identifying pollen returned to the hive provides a direct measure of pollen foraging, whilst the pollen within honey provides a longer-term overview of plants being used for both nectar and pollen [29–31]. These methods are typically used to identify the botanical composition of honey in order to check its geographic origin for food quality and traceability purposes [29]. They have more rarely been used to investigate foraging preferences [32].

The traditional method for identification of honey bee collected pollen is through morphological features revealed with light microscopy. If the pollen is within honey the extraction and identification of the pollen is termed melissopalynology [29]. Morphological identification of pollen requires considerable skill and experience [33]. Some plants can be particularly difficult to distinguish, for example species of Campanulaceae, Lamiaceae and Poaceae, as these exhibit few unique morphological features [34–36]. Within the Rosaceae individual species can show high levels of pollen grain morphological variation, making characterisation difficult [37].

The use of DNA-based methods for pollen identification has attracted interest in recent years. DNA-based identification has the potential to reduce processing time and increase the level of species discrimination. In addition, it does not require the high level of taxonomic expertise required for microscopic examination [33, 38–40]. DNA can be successfully extracted from honey bee pollen loads and from within honey using a range of extraction methods [40–43]. A number of techniques have been used to identify the plant species. Laube et al. (2010) successfully used real-time PCR to identify plant species found within Corsican honey, but this method requires an *a priori* knowledge of the species likely to be found [44]. Wilson et al. (2010) investigated pollen foraging in Hawaiian *Hylaeus* bees using PCR followed by Sanger sequencing; this is effective for samples composed of a single plant species but cannot be used for pollen containing a mixture of species [45]. PCR amplification, followed by cloning and sequencing has been used for both pollen [35] and honey [33, 38, 46]. Cloning is a time consuming process, however, and places limits on the sequencing depth that can be obtained.

A DNA metabarcoding approach combining the amplification of universal markers with high throughput sequencing (HTS) gives the opportunity to analyse many samples, containing mixtures of species, with extensive depth of coverage [47]. HTS technologies, especially Roche 454 and Illumina sequencing platforms, have been successfully used in applications including the composition of microbial [48, 49], freshwater [50] and fungal communities [51, 52], diet analysis [53–55] and biodiversity assessments [56, 57].

A small number of studies have investigated the use of pollen DNA metabarcoding. Kraaijeveld et al. (2015) used the Ion Torrent system to assess the effectiveness of DNA metabarcoding for quantifying airborne pollen, whilst Richardson et al. (2015) used Illumina Mi-seq and Keller et al. (2015) used 454 pyrosequencing to characterise pollen retrieved from pollen traps on honey bee hives [36, 58, 59]. Valentini et al. (2010) trialled the use of 454 pyrosequencing to characterise two commercial honey samples [39].

Previous studies have used either plastid markers such as *rbcL*, *trnH-psbA* and *trnL* [33, 35, 36, 38, 39] or the nuclear ITS and ITS2 region [45, 58, 59]. Key considerations for markers used for DNA metabarcoding are: 1) Primers should be universal so that all of the species within the mixture are amplified. 2) The region should have a level of discrimination suitable for the questions being asked. 3) Correct identification relies on the quality and comprehensiveness of the reference library that unknown samples are compared to [60].

*rbcL* is one of the internationally agreed core DNA barcodes for plants [61], whilst *trnH-psbA* and ITS2 are recognised as valuable additional markers [62–64]. ITS2 and *trnH-psbA* often exhibit higher levels of species discrimination compared to *rbcL* but they have lower levels of universality [63,64]. This lower universality means that some species within mixed samples will not be detected using ITS2 and *trnH-psbA* [60].

In this study we assess the potential of using DNA metabarcoding to characterise the floral composition of honey in order to investigate honey bee foraging. We compare DNA metabarcoding using the *rbcL* DNA barcode marker and 454 pyrosequencing with microscopic analysis for nine honey samples provided by domestic beekeepers in Wales and England (UK). We then use the plants recorded with both techniques to discuss honey bee foraging preferences.

We have used the *rbcL* marker as it is one of the core DNA barcodes for plants and provides the highest degree of universality of all the regions assessed for DNA barcoding [61]. de Vere *et al*. (2012) have assembled a *rbcL* DNA barcode reference library for 98% of the native Welsh flora as part of the Barcode Wales project [65]. This provides a comprehensive reference library for this current study.

## Materials and Methods

### Honey Samples

**Fig 1:**
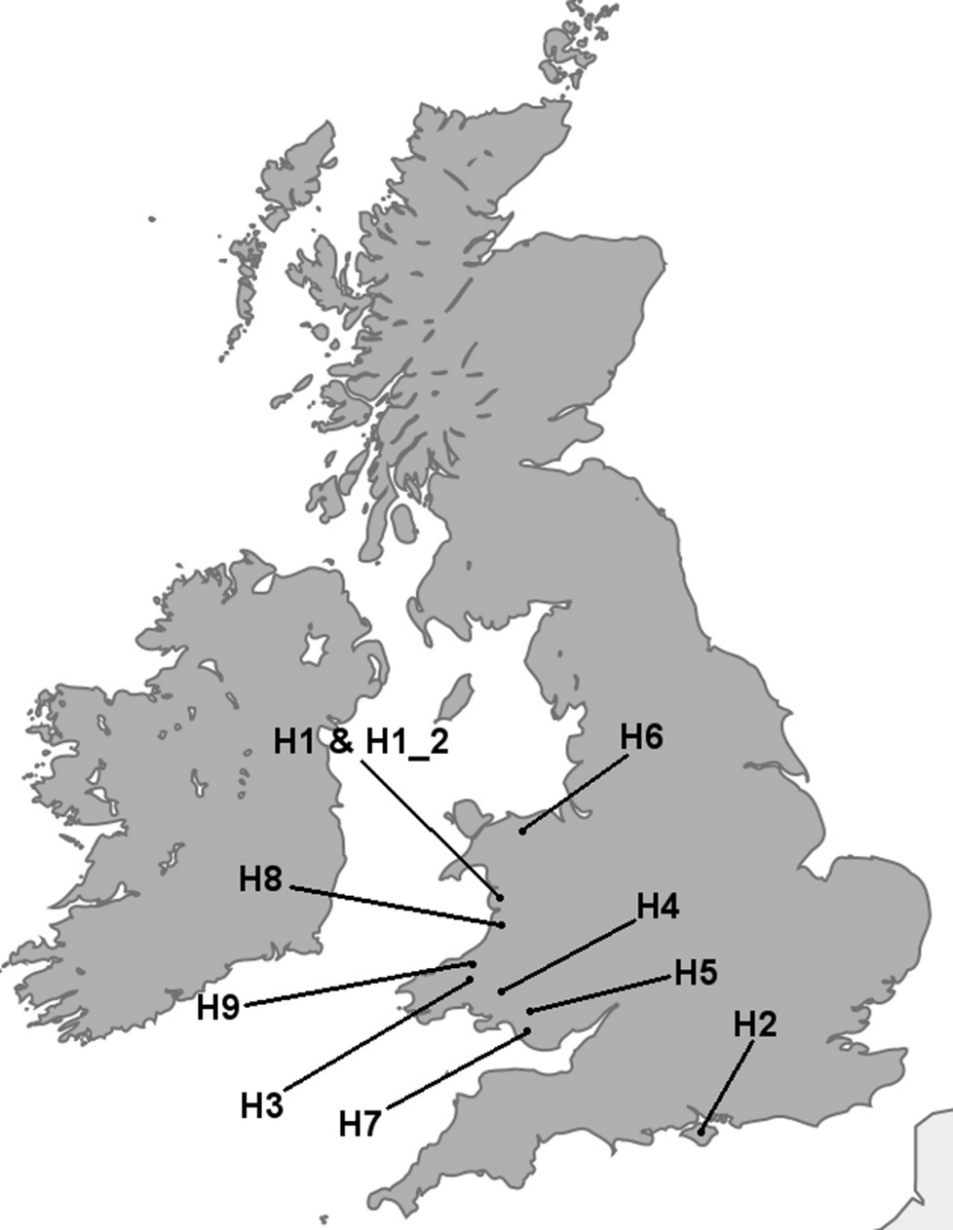
The location of the honey bee colonies from which honey was provided by domestic beekeepers.

Honey samples were provided by domestic beekeepers from Wales and England (UK) (Table 1 and Fig 1). Nine different colonies were sampled. For one colony, two samples from the same hive (Honey H1 and H1_2), were analysed to provide a measure of repeatability. The hives were located in gardens or small-holdings and descriptions from the beekeepers and examination of aerial images using Google Earth were used to describe the dominant vegetation types surrounding the hives. An area with a radius of 7km from the location of the hives was examined (Table 1). This distance was taken as a realistic foraging range based on a number of foraging studies on honey bees [23–27].

S1 File provides the hive locations as a KML (Keyhole Markup Language) file loadable in Google Earth.

**Table 1:** The locations of the honey samples analysed using DNA metabarcoding and melissopalynology.

Honey	Vegetation surrounding hive (7km radius)	Coordinates (Lat/Long)
H1 & H1_2	Small town with many gardens, close to the sea, riparian, semi-improved and improved pasture.	52.587166, -4.083243
H2	Woodland, oil seed rape, semi-improved and improved pasture. Close to the sea with towns nearby.	50.735613, -1.269290
H3	Woodland, riparian, semi-improved and improved pasture.	52.002253, -4.413797
H4	Improved pasture and riparian.	51.876283, -4.105855
H5	Woodland, riparian, semi-improved and improved pasture, moorland.	51.776733, -3.534182
H6	Improved pasture.	53.171426, -3.610821
H7	Woodland, semi-improved and improved pasture, riparian. Close to a town.	51.564926, -3.602989
H8	Woodland, semi-improved and improved pasture, riparian. Large plots of vegetables and fruit trees. A large town nearby.	52.366529, -4.050206
H9	Woodland, semi-improved and improved pasture.	52.071726, -4.387493

Honey samples were provided by domestic beekeepers from hives located in gardens or smallholdings. The vegetation surrounding the hives was characterised based on descriptions from the beekeepers and observation of aerial images. H1 and H1_2 were two samples taken from the same hive.

### Melissopalynology

Pollen analysis was performed following the guidelines of the International Honey Commission [29, 66]. 2 g of honey was mixed with 40 ml of 0.5% sulphuric acid solution. Samples were incubated in a water bath for 5 minutes at 80°C. Samples were filtered through a 5 μm filter, placed in a filter assembly with pump, and 500 ml hot distilled water was used to rinse the samples. The filter was washed using 8 ml of glacial acetic acid to dehydrate the sample and a centrifuge step was performed at 3000*g* for 2 minutes. The supernatant was decanted, mixed with 1 ml acetolysis mixture and placed in a water bath at 80°C for 12 minutes. The centrifugation step was repeated, the sample was then resuspended in 1 ml glacial acetic acid and centrifuged once more. Three drops were mounted onto a microscope slide and examined under a light microscope at x400 and x1000 magnification. Identification of the pollen grains was undertaken with reference to type slides of pollen from the United Kingdom and to pollen atlases [67–70]. A minimum of 300 grains were characterised and unidentifiable grains were also noted.

### DNA extraction

Total DNA was extracted from 10 g of honey using a protocol developed from published methods adapted for use within the current study [38, 39, 41, 42, 44, 71]. Total DNA from each of the honeys was extracted four times so that DNA was recovered from a total of 40 g of each honey. For each DNA extraction the honey sample was placed into a sterile 50 ml centrifuge tube followed by the addition of 30 ml of ultrapure water. Samples were incubated at 65°C for 30 minutes with occasional shaking. Samples were then centrifuged for 30 minutes at 15,000*g*. The supernatant was discarded and each pellet was resuspended in 400 μl of Buffer AP1 from a DNeasy Plant Mini Kit (Qiagen) to which 80 μl of proteinase K (1 mg/ml) had been added. Samples were disrupted using a TissueLyser II (Qiagen) with 3 mm tungsten carbide beads for 4 minutes at 30 1/s and then incubated for 10 minutes at 65°C in a waterbath. The subsequent steps of the DNeasy Plant Mini Kit were followed according to the manufacturer’s instructions, with the exception that the QIAshredder column and second wash stage were omitted. The extracted DNA was stored at -20°C prior to subsequent analysis.

### Amplification and Sequencing

DNA was amplified using the *rbcL* DNA barcode marker region [61]. Two rounds of PCR were carried out, firstly to amplify the *rbcL* region and then to attach unique 5 bp tags so that different samples could be separated bioinformatically after sequencing.

Samples were first amplified using the universal primers rbcLaf and rbcLr590 [65] to which adaptor ‘tails’ had been added (rbcLaf+adaptor: GACGATGAGTCCTGAGGTATGTCACCACAAACAGAGACTAAAGC rbcLr590+adaptor: GACGATGAGTCCTGAGGTAGTCCACCGCGTAGACATTCAT). PCR was performed in a final volume of 25 μl. A total of 2.5 μl of template DNA was combined with 12.5 μl of 2x Biomix (Bioline), 0.5 μl of each primer (5 μM), 1.0 μl of BSA (10 μM) and 8.0 μl of molecular grade water (Sigma-Aldrich). The reaction was performed in a thermal cycler MJ Mini (Bio-Rad Laboratories, Hercules, CA, USA) using the following program: initial denaturing at 95°C for 2 minutes, followed by 30 cycles of 95°C for 2 minutes, 50°C for 90 seconds, 72°C for 40 seconds with a final extension at 72°C for 5 minutes and 30°C for 10 seconds [65].

The products were visualised on a 1% agarose gel and those producing the brightest bands were diluted by 1/2000, those producing medium bands were diluted by 1/1000 and those producing faint bands were diluted by 1/500. 2.5 μl of the diluted product was used as the template for the second round of PCR. This contained a mixture of 12.5 μl of 2x Biomix (Bioline), 1.0 μl of a unique 5 bp tag with adaptor (10 μM), 1.0 μl of BSA (10 μM) and 8.0 μl of molecular biology grade water. The PCR reaction was repeated as for the first PCR but with 15 cycles. Each sample was amplified twice using this procedure and the resulting products from all the PCR runs were then pooled and purified using a QIAquick PCR purification kit (Qiagen). A total of 90 μl of pooled DNA was sent for Roche/454 GS FLX Titanium pyrosequencing at the University of Pennsylvania using a ¼ plate.

### Data Analysis

Sequences were sorted according to the identity of their 5 bp tag into the different honey samples and were assessed for quality and length. Any sequences where the 5 bp tag and the entire primer sequence could not be found were removed. The tag and primer sequences were then trimmed and sequences with a read length of 250 bp or less after trimming were discarded.

Two local BLAST databases were created; the first was from *rbcL* sequences obtained from the Barcode Wales project, which provided 98% coverage for the native flowering plants of Wales [65]. The second database was generated by extracting all of the chloroplast sequence data from GenBank. Each DNA sequence obtained from the honey samples was scored simultaneously against both databases using Megablast. If the sequence top bit score matched to a single species, then the sequence was identified to that species. If the top bit score was the same for different species belonging to the same genus, then the result was given to genus. If the top bit score belonged to multiple genera within the same family then a family level designation was made. Sequences blasting to multiple families were considered to be unknown. Scripts written in Python were used to automat the BLAST analysis and to summarise the output (S2 File).

Results for each honey sample were then manually filtered so that only species recorded within the UK were retained. Stace (2010) and Cubey & Merrick (2014) were used as references for plants occurring in the UK as natives, aliens or in horticulture or agriculture [72, 73]. If a species was not recorded within the UK then the sequence was reclassified to genus level.

To reduce results arising from amplification or sequencing errors, taxa recorded from less than 10 sequences for that honey sample were removed from further analysis [60]. Files containing the sequence reads used in this study are available through the sequence read archive (http://www.ncbi.nlm.nih.gov/Traces/sra/?study=SRP055687, SRA accession: SRP055687).

## Results

### Comparison of DNA metabarcoding and melissopalynology

In total 51,131 sequences over 250 bp in length could be attributed to tagged sequences of *rbcL* using the 454 pyrosequencing approach (Table 2). Of these, 47,512 reads (93%) could be characterised to family, genus or species level. One sample, H3, provided a very low number of reads that could be assigned (149) but for the other honeys the number of identifiable reads ranged from 3745 to 8097 (Table 2). Sequence quality was also slightly lower for sample H3. For the other samples the results were very similar with an average QV of 28 and with between 83% to 85% of reads having a QV greater than 20. The number of pollen grains counted using melissopalynology was fixed at around 300 grains per sample. Of these, a high percentage could be identified to species, genus or family level (98% to 100%).

**Table 2:** Number of pollen grains and DNA sequences analysed, their quality and the number that were successfully identified to family, genus or species level.

Honey	DNA metabarcoding					Melissopalynology	
	DNA reads >250 bp, tags present	Number identified to family, genus or species level (%)	Mean Length DNA (SD)	Mean QV DNA (SD)	Mean % reads with QV >20 DNA	Pollen grains counted	Number identified to family, genus or species level (%)
H1	4612	3976 (86)	389 (72)	28 (2)	84 (6)	334	334 (100)
H2	6862	6505 (95)	387 (70)	28 (2)	84 (6)	317	317 (100)
H3	168	149 (89)	328 (60)	26 (2)	80 (7)	338	330 (98)
H4	4325	4202 (97)	388 (71)	28 (2)	85 (6)	367	361 (98)
H5	8564	8097 (95)	376 (71)	28 (2)	84 (6)	309	303 (98)
H6	3922	3745 (95)	385 (71)	28 (2)	84 (7)	293	290 (99)
H7	7575	6851 (90)	383 (71)	28 (2)	84 (6)	346	339 (98)
H8	5168	4619 (89)	391 (69)	28 (2)	84 (6)	206	201 (98)
H9	4649	4429 (95)	387 (70)	28 (2)	83 (7)	306	304 (99)
H1_2	5286	4939 (93)	385 (72)	28 (2)	83 (7)	364	356 (98)

A total 46 plant families from 25 orders were recorded from honeys H1 to H9 using DNA metabarcoding and melissopalynology (S1 Dataset). With melissopalynology the number of taxa detected at family, genus or species level ranged from 8 to 31 whilst DNA metabarcoding detected 5 to 24 taxa across the honey samples (Table 3 and S1 Dataset). On average DNA metabarcoding identified a greater number of the taxa to species level (31% compared to 27%) but this difference was not statistically significant (t: 1.288, df: 16, p: 0.216). Both DNA metabarcoding and melissopalynology detected most taxa to genera (mean 51% and 50% respectively).

**Table 3:** The number of taxa detected within honey samples using DNA metabarcoding and melissopalynology, and the proportion of these identifiable to family, genus or species level (%).

	DNA metabarcoding				Melissopalynology				Both combined			
	Taxa (n)	Family %	Genus %	Species%	Taxa (n)	Family %	Genus %	Species %	Taxa (n)	Family %	Genus %	Species %
**H1**	24	21	54	25	31	13	61	26	45	16	60	24
**H2**	18	11	61	28	8	25	63	13	20	15	55	30
**H3**	5	20	40	40	17	35	35	29	17	35	35	29
**H4**	12	17	42	42	12	17	50	33	19	16	53	32
**H5**	18	28	50	22	16	13	56	31	27	26	48	26
**H6**	10	20	50	30	13	38	38	23	17	35	41	24
**H7**	21	19	43	38	20	25	45	30	32	25	47	28
**H8**	19	21	63	16	10	30	40	30	20	20	60	20
**H9**	11	9	55	36	9	33	44	22	15	27	47	27
**Mean**	15	18	51	31	15	25	48	26	24	24	50	27
**SD**	6	6	8	9	7	9	10	6	10	8	8	4

**Fig 2:**
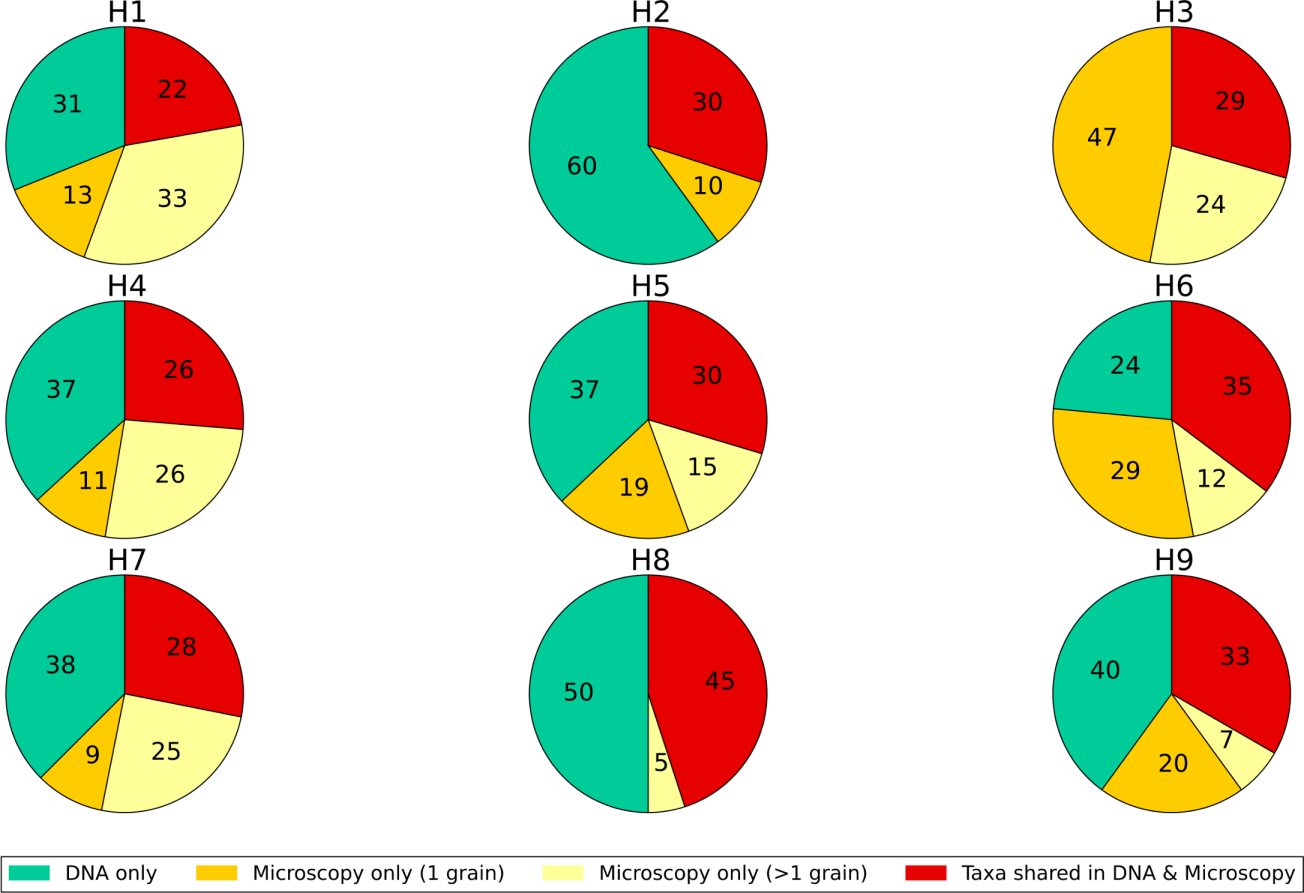
The similarity of plant taxa found in nine honey samples using DNA metabarcoding and melissopalynology. The number of taxa detected (at family, genus or species level) is divided into those found with both techniques and those found using only one method. The taxa found using melissopalynology only are further subdivided into those where multiple pollen grains were found and those characterised with just a single grain. The values in the pie chart are the % of taxa within each category.

The number of taxa that match between DNA metabarcoding and melissopalynology ranges from 22% to 45% (Fig 2). Both DNA metabarcoding and melissopalynology detect additional taxa not found with the other technique. This ranges from 24% to 60% additional taxa for DNA metabarcoding and from 10% to 46% for melissopalynology. For most of the honeys (6 out of 9) DNA metabarcoding detects more additional taxa compared to melissopalynology. A high proportion of the taxa recorded using microscopy but not found in the DNA analysis were represented by single pollen grains, indicating that they are at low abundance within the honey sample (Fig 2).

There are 11 plant families with more than one genus or species recorded for either DNA metabarcoding or microscopy (Table 4). Of these, five families have the same number of taxa detected for DNA and microscopy. However, for the Rosaceae, Asteraceae and Onagraceae, DNA metabarcoding identifies a greater number of taxa. For the Boraginaceae, Ranunculaceae and Euphorbiaceae, microscopy detects more taxa but each of these is only represented by one or two pollen grains (Table 4 and S1 Dataset). The proportion of taxa distinguishable to species is similar with both methods. Over all of the families, DNA metabarcoding and melissopalynology each detect 26 taxa. DNA metabarcoding is able to distinguish 10 out of 26 to species level compared to 8 out of 26 using microscopy.

**Table 4:** Number of taxa detected within each family across nine honey samples analysed by DNA metabarcoding, melissopalynology and both techniques combined. Only families containing more than one taxon are considered.

Family	DNA metabarcoding			Melissopalynology			Melissopalynology and DNA metabarcoding		
	Genus	Species	Total	Genus	Species	Total	Genus	Species	Total
Rosaceae	5	2	7	3	2	5	6	2	8
Asteraceae	3	2	5	2	1	3	4	2	6
Fabaceae	3	0	3	3	0	3	4	0	4
Boraginaceae	0	0	0	1	1	2	1	1	2
Betulaceae	0	2	2	1	1	2	1	2	3
Fagaceae	1	1	2	1	1	2	1	1	2
Asparagaceae	1	1	2	1	1	2	1	1	2
Onagraceae	1	1	2	1	0	1	1	1	2
Ranunculaceae	1	0	1	2	0	2	2	0	2
Sapindaceae	1	1	2	1	1	2	1	1	2
Euphorbiaceae	0	0	0	2	0	2	2	0	2
Total	16	10	26	18	8	26	24	11	35

**Fig 3:**
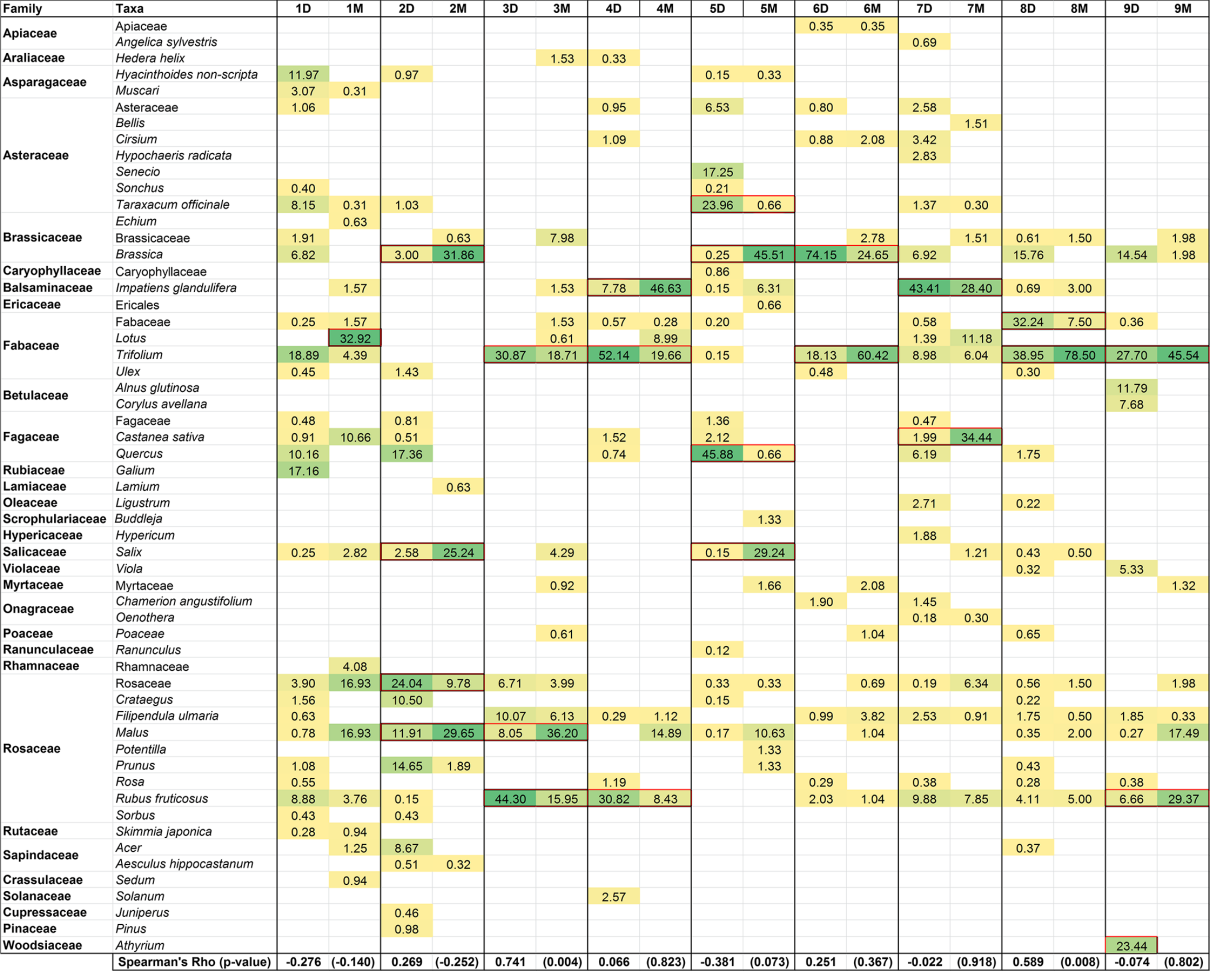
Plants identified using DNA metabarcoding (D) and melissopalynology (M) for nine honeys. Values represent the percentage of the total number of pollen grains or sequence reads obtained for that honey. Taxa recorded using microscopy only based on the presence of a single pollen grain are not shown (these are provided in full in S1 Dataset). Boxes outlined in red denote taxa with an abundance of 20% or over for either technique. Correlations between the relative abundance of taxa recorded with the two methods are assessed with Spearman Rank Correlations. Spearman’s Rho is shown with the corresponding p-value in brackets.

Although there are differences in the plants found between the techniques, the dominant floral components of the honeys are detected with both methods. However, the relative abundance detected differs. There are 24 taxa (family, genus or species level) that appear with >20% abundance across all of the honey samples using both DNA metabarcoding and microscopy (indicated with a red outline in Fig 3). Of these, 22 taxa are found using both methods, giving 92% correspondence between DNA and microscopy. Of the two plants that do not match, one of these is DNA from a fern that would not have been included in the microscopic analysis. The relative abundance of the taxa within the honey samples using the two methods generally does not correlate (Fig 3). After Bonferroni correction for multiple testing, only one honey (H3), shows a significant correlation between the abundance of taxa found using the two techniques.

### Repeat sampling using DNA metabarcoding and melissopalynology

DNA metabarcoding and melissopalynology was repeated on two samples of honey H1 (H1 and H1_2) in order to investigate repeatability. Each sample was a separate extraction from the same hive collected at the same time. The two methods detected comparable numbers of taxa within each repeat but the similarity of the taxa found was much higher for DNA metabarcoding compared to melissopalynology (Table 5 and S2 Dataset). There was a 64% match of the taxa found using DNA metabarcoding compared to just 28% with melissopalynology.

**Table 5:** Similarity of taxa (family, genus or species) detected for honey sample H1 and H1_2. These are two different extractions from the same hive, collected at the same time.

	**DNA metabarcoding**		**Melissopalynology**	
	H1	H1_2	H1	H1_2
Number of sequence reads or pollen grains	3976	4939	326	340
Number of taxa (family, genus or species)	24	22	31	28
Total number of taxa detected	28		46	
Number of taxa shared between H1 and H1_2	18		13	
Similarity of H1 and H1_2 (%)	64		28	

### Plants used by honey bees for foraging

A number of plants were recorded from more than one honey sample (Table 6). The most frequently found herbaceous plants were *Rubus fruticosus*, *Filipendula ulmaria*, *Impatiens glandulifera*, *Taraxacum officinale* and species belonging to *Trifolium* and *Brassica* (Table 6 and Fig 3). Tree pollen was also frequently seen, especially *Castanea sativa*, *Crataegus monogyna*, along with *Malus*, *Salix* and *Quercus* species (Table 6).

**Table 6:** Species and genera found in more than one honey sample for DNA metabarcoding and melissopalynology.

**Family**	**Taxa**	**DNA metabarcoding: number of honeys**	**Melissopalynology: number of honeys**
**Identified to species**			
Rosaceae	*Rubus fruticosus*	8	7
Rosaceae	*Filipendula ulmaria*	7	6
Fagaceae	*Castanea sativa*	5	2
Balsaminaceae	*Impatiens glandulifera*	4	7
Asteraceae	*Taraxacum officinale*	4	4
Rosaceae	*Crataegus monogyna*	4	0
Asparagaceae	*Hyacinthoides non-scripta*	3	1
Onagraceae	*Chamerion angustifolium*	2	0
Araliaceae	*Hedera helix*	1	2
Aquifoliaceae	*Ilex aquifolium*	0	2
**Identified to genus**			
Fabaceae	*Trifolium*	8	7
Brassicaceae	*Brassica*	7	4
Rosaceae	*Malus*	6	9
Fagaceae	*Quercus*	6	1
Rosaceae	*Rosa*	6	0
Salicaceae	*Salix*	4	7
Fabaceae	*Ulex*	4	0
Rosaceae	*Prunus*	3	2
Asteraceae	*Cirsium*	3	1
Sapindaceae	*Acer*	2	3
Oleaceae	*Ligustrum*	2	1
Asteraceae	*Sonchus*	2	0
Violaceae	*Viola*	2	0
Rosaceae	*Sorbus*	2	0
Fabaceae	*Lotus*	1	5
Ranunculaceae	*Ranunculus*	1	3
Asteraceae	*Bellis*	0	2
Betulaceae	*Betula*	0	2

An interesting observation was the presence of non-flowering plants detected with the DNA analysis but not microscopy. *Juniperus* and *Pinus* species were found in honey H2, and DNA from the fern genus *Athyrium* was found in high levels in honey H9 (Fig 3).

The plants detected reflect the vegetation that surrounds the hives. The high frequency of *Trifolium* is indicative of the improved and semi-improved pastures found close to all of the hives, and *Taraxacum officinale* and *Rubus fruticosus* are frequent in the rough grassland and hedgerows surrounding pasture. *Impatiens glandulifera* is a highly invasive, non-native species, of riparian habitat and is consistently found when the hives are close to rivers. *Filipendula ulmaria* is a common species of damp grasslands and hedgebanks and is found in all of the Welsh honeys but not in the honey from England (H2).

H2 was the only sample from hives located close to fields of oil seed rape. The high level of *Brassica* pollen in this honey is therefore likely to be *Brassica napus*. Interestingly, though, many of the honeys contain *Brassica* pollen, although the other hives are not located near to oil seed rape. The *Brassica* detected in these samples may be garden *Brassica* species grown for food or the commonly occurring *Brassica rapa* [74].

Three of the honey samples came from hives in the vicinity of urban areas (H2, H7 and H8) but only one (H1) had hives located within an urban area containing many small gardens. This honey has more taxa than the other honey samples and features a range of horticultural plants including species belonging to *Muscari*, *Galium*, *Rosa*, *Prunus*, *Sorbus* and *Skimmia japonica* (S1 Dataset).

## Discussion

### Using DNA metabarcoding and melissopalynology for identifying the floral composition of honey

DNA metabarcoding and melissopalynology are both effective methods for detecting the most abundant pollen found within honey samples. Both methods detect the dominant constituents of honey but show differences in some of the plants and the relative abundance of these found within the honey sample. This is not surprising as honey is a highly heterogeneous natural product and the sampling strategy adopted for the two methods is different. Melissopalynology uses a starting sample of 2 g of honey whilst the DNA method adopted here uses 40 g of honey in total.

Analysis of honey H1 and H1_2 that came from the same hive reflects the variability of sampling. DNA metabarcoding has a much higher reproducibility, with 64% similarity compared to 28% for melissopalynology. This is likely to be due to the greater amount of pollen that can be screened using the DNA metabarcoding approach.

The level of similarity in the plants detected with the two techniques ranges from 22% to 45%. Honey appears to contain a fairly small number of dominant constituents and then a longer list of plants found in much smaller amounts. Multiple sampling of the same honey with the construction of species accumulation curves would be required to fully characterise this long tail of species with low abundance [75]. The degree of characterisation required, however, depends on the question being posed. For many questions, determining the most frequently used floral resources is of more relevance than determining the less frequently used plant species.

Some of the differences in the plants detected reflect biases within both the DNA metabarcoding and microscopic analysis. For some families, for example the Rosaceae and Asteraceae, DNA metabarcoding provides a higher level of resolution compared to melissopalynology. For other families such, as the Boraginaceae and Euphorbiaceae, DNA metabarcoding appears less able to detect species within these groups.

DNA metabarcoding and melissopalynology show little correspondence in the relative abundance of taxa found within the honey samples. As a direct count of the pollen grains present, melissopalynology provides a more quantitative measure, albeit with a smaller sample size. The inability of DNA metabarcoding to provide quantitative results has been observed in a range of studies [36, 53, 56, 58]. There are many stages in the DNA analysis process where biases can occur that will prevent a quantitative estimation of floral composition.

Pollen grains vary in shape, size and pollen wall composition and this is likely to affect DNA extraction efficiency for different species [76]. The DNA analysed for DNA metabarcoding often comes from plastid markers, such as *rbcL*, *trnL* or *psbA-trnH* [33, 35, 36, 39, 55]. Within most flowering plants chloroplasts are maternally inherited but the pollen grain contains plastids within the vegetative cell that can be targeted. The number of plastids varies with different species and also with the maturity of the pollen grain [76]. Nevertheless, studies on DNA extracted from pollen have shown excellent ability to amplify plastid markers over a wide range of species, confirming the presence of sufficient DNA [33, 35, 36, 39].

Once the DNA is extracted, PCR biases can lead to some taxa being preferentially amplified [77]. In order to minimise this it is important to use a marker with a high degree of universality across a broad range of taxonomic groups [78]. We have used the *rbcL* DNA barcode marker as it has been shown to have the highest universality of all markers that have been proposed for DNA barcoding plants [61]. However, even with high universality of primers, when working on species in isolation, amplification may still be skewed within a multi-template PCR [53]. Some species, especially those present in low quantities, can be missed out when a mixed sample is amplified [57, 79]. This is illustrated within the current study where species recorded using microscopy from a single pollen grain are less likely to be detected using DNA metabarcoding. Techniques are being developed that avoid the PCR stage altogether, such as using shotgun sequencing with subsequent recovery of DNA barcode markers or even whole chloroplasts [56, 78, 80, 81].

A key factor in the ability to identify species using DNA metabarcoding is the comprehensiveness and quality of the reference database that unknown sequences are compared to [78]. This study relies on the fact that 98% of the Welsh native flora has been DNA barcoded with the *rbcL* region [65]. This library is not complete, however, as none of the non-native and garden flora have been DNA barcoded, meaning that identifications for these species rely on the availability of these groups in GenBank.

Groundtruthing using known distributions of plant species for the geographic area being sampled can help to improve discrimination [65]. The method used here filters the BLAST results generated so that only plants found growing within the UK, whether native, alien or in horticulture or agriculture, are recorded, with any remaining listed as unknown.

Melissopalynology as a method for characterising the floral composition of honey also has limitations. Some plant groups are known to be difficult to distinguish and some plants can only be taken to the level of family due to lack of morphological differences in the pollen grains [35, 36]. The major limitation to the microscopic investigation of honey, however, is the high degree of expertise required to identify the different pollen types. The time associated with identifying each pollen grain puts limitations on the size of sample that can be screened. Microscopic analysis provides a direct quantification of the number of pollen grains but because only a limited number of pollen grains can be processed it means the coverage of the honey is low.

Although both DNA metabarcoding and melissopalynology have limitations, both are useful tools in understanding the floral composition of honey. The value of DNA metabarcoding is that a greater volume of honey can be investigated and the technique does not require the high level of taxonomic expertise required to identify the pollen within the honey using microscopy. Once optimised, the technique allows the identification of many samples very quickly. It is not suitable, however, if an exact estimation of pollen quantity is required, and it is possible that species occurring at low levels will not be detected.

DNA metabarcoding has the potential to provide a valuable technique for identifying the floral composition of honey relevant to ecological studies investigating foraging and also food quality assessments requiring an understanding of the geographic source of honeys [82, 83]. The technique can easily be adapted to survey pollen found on the bodies of other pollinating insect groups, providing a valuable method for assessing pollinator foraging preferences.

### Using the floral composition of honey to investigate honey bee foraging

Examining the floral composition of honey provides a means of observing honey bee foraging over the season in which the honey was made. The honeys examined here cover a geographic range of 315 km and encompass both agricultural and more urban habitats. The floral composition of honey reflects the environment the honey bees are foraging within. *Trifolium* species are characteristic of improved and semi-improved pastures, whilst *Taraxcum officinale*, *Rubus fruticosus* and *Filipendula ulmaria* are found in hedgebanks and rough grassland. The invasive *Impatiens glandulifera* is seen where hives are located within foraging range of rivers. The frequent presence of *Malus* and *Rosa* species reflects the location of hives in the gardens and small-holdings of domestic beekeepers where these plants are frequently grown. *Brassica* species are regularly detected and are likely to be either oil seed rape (*Brassica napus*), garden *Brassica* species grown for food or the commonly occurring *Brassica rapa* [74].

Of particular note is the greater number of plant species recorded when the honey bees are located within a small urban area with many gardens, where a range of commonly grown garden plants are detected (H1). The importance of gardens for honey bees and wild pollinators is being increasingly recognised [15–19]. Lists of ‘pollinator-friendly’ plants are widely available but often do not have a firm evidence base [20]. Using DNA metabarcoding of honey provides a method for assessing the horticultural plants honey bees actually use. This approach can be adapted for other pollinator groups such as bumblebees, solitary bees and hoverflies by DNA metabarcoding the pollen on their bodies, in order to assess the plants they visit. This can be used to refine pollinator plant lists in order to provide more targeted advice to gardeners, farmers and landowners.

Although the plants reflect the vegetation the honey bees are foraging within, what is especially notable is the similarity of the plants found across the different honey samples. Honey bees are generally considered to be supergeneralists, utilising a very wide range of plant species for nectar and pollen [2, 84]. The honeys examined in this study contained plants with a broad taxonomic range, covering 46 families and 25 orders, but a relatively small number of plants are consistently recorded across the majority of the honeys. The plants detected here will be of no surprise to beekeepers, who have long stated that particular plants are important to honey bees throughout the season [85]. Synge (1947) used pollen trapping to produce a detailed list of plants foraged upon by honey bees located in the Rothamsted Experimental Station in SE England [30]. Of the 34 major pollen sources he notes, we find 62% of these in our honey samples. He describes 54 plants of minor importance and we have detected 33% of these. Free and Williams (1974) investigated floral constancy of honey bees placed in crops of sweet cherry, kale, field bean and red clover within the Rothamsted Experimental Station [86]. Although these crops provided an abundant nectar and pollen source, and were actively worked by the honey bees, they also regularly utilised a small number of additional plants. Of the 15 commonly used plants recorded by Free and Williams (1974), 11 (73%) are recorded within the present study including the frequently observed *Rubus fruticosus*, *Filipendula ulmaria*, *Taraxacum officinale*, *Crataegus monogyna*, *Trifolium* and *Brassica* [86]. A valuable area of further research is to discover why these particular species are important. This could relate to the ease of availability and abundance of the plant, the quality and abundance of the nectar and pollen and/or specific nutrients or trace elements provided by these species. An understanding of the reasons why honey bees target certain plants could help to provide guidance on what constitutes a balanced honey bee diet.

Along with the frequently found species there are some interesting plants found only in one or two honey samples. The presence of non-flowering species such as juniper and pine may reflect honey bees collecting resin from these conifers in order to make propolis [87]. Resin is actively collected from a range of species and combined with wax to make propolis which is deposited within the hive as it has antimicrobial properties [87]. Another possibility is that the presence of DNA of conifers is due to bees foraging on honeydew. Honey bees sometimes collect the exudate from sap-sucking insects as an alternative to nectar [88].

An unusual plant recorded here is DNA belonging to the fern genus *Athyrium*. This occurs in just one honey sample so could potentially be considered as an anomalous result, but *Athyrium* was also recorded in a commercial honey analysed by Valentini (2010)[39]. We cannot find any discussion in the literature of honey bees collecting material from pteridophytes, but the presence of pteridophyte spores has been recorded in a small number of palynological investigations from Nigeria [89], Nepal [90] and New Zealand [91]. The spores are typically considered to be a contaminant that has occurred at some stage in the processing of the honey. But spores are a protein source much like pollen so honey bees may well forage upon them. Although the collection of fern spores has not been discussed, honey bees collecting fungal spores was first recorded by Cook in 1885 and sporadically in other reports since then [92]. Along with discovering the reasons why some plants are consistently used by honey bees, understanding more about the rarely recorded species may reveal interesting insights into honey bee behaviour.

Honey DNA metabarcoding and melissopalynology provide valuable techniques for recording the plants honey bees visit for nectar and pollen. This provides a starting point for investigating what plants are most important within the honey bees environment in order for them to meet their nutritional needs and maintain healthy colonies. If certain species or types of plant are favoured throughout the year then we can ensure that these are provided.

Both DNA metabarcoding and melissopalynology are effective techniques for detecting the most abundant floral sources within honey, but DNA metabarcoding has the advantage of not requiring high levels of taxonomic expertise. It provides a method that can be easily and widely used to answer questions about pollinator foraging behaviour.

## Supporting Information

S1 Dataset Full list of taxa detected for nine honeys using DNA metabarcoding and melissopalynology. (XLSX)

S2 Dataset Full list of taxa detected for two honey samples, collected from the same hive, and analysed using DNA metabarcoding and melissopalynology. (XLSX)

S1 File Compressed KML file, loadable in Google Earth, showing the location of nine honey bee colonies. (ZIP)

S2 File Python scripts used to analyse DNA metabarcoding data. (ZIP)
