# Supplementary material for: Using DNA Metabarcoding to Identify the Floral Composition of Honey: A New Tool for Investigating Honey Bee Foraging Preferences
Source: PLoS One. 2015 Aug 26;10(8):e0134735. doi: 10.1371/journal.pone.0134735 (PMC4550469; doi:10.1371/journal.pone.0134735)
Supplement: S2 File — (ZIP) [file pone.0134735.s004.zip › BLAST-Tools/blast-db/Readme.rtf]

The local BLAST databases to be used should be placed in here.
